# Unmet need in Sierra Leone: a national oral health survey of schoolchildren

**DOI:** 10.1038/s41405-022-00107-7

**Published:** 2022-06-14

**Authors:** S. G. Ghotane, S. J. Challacombe, P. Don-Davis, D. Kamara, J. E. Gallagher

**Affiliations:** 1 Department of Women & Children’s Health, Faculty of Life Sciences & Medicine, King’s College London, Becket House, 1 Lambeth Palace Road, London, SE1 7EU UK; 2 Faculty of Dentistry, Oral & Craniofacial Sciences, Centre for Host Microbiome Interactions, King’s College London, Floor 22, Guys Tower, Guys Hospital, London, SE1 9RT UK; 3 College of Medicine and Allied Health Sciences, Connaught Hospital, Freetown, Sierra Leone; 4 Oral Health Department, Connaught Hospital, Freetown, Sierra Leone; 5 Faculty of Dentistry, Oral & Craniofacial Sciences, King’s College London, Centre for Host Microbiome Interactions, Denmark Hill Campus, Bessemer Road, London, SE5 9RS UK

**Keywords:** Dental epidemiology, Oral diseases

## Abstract

**Objective:**

Sierra Leone (SL), in West Africa, with a population of over 7.5 million people has suffered the effects of a civil war previously, and more recently Ebola & Covid-19. Dental care is very limited, mostly in the capital Freetown and the private sector. No dental education is available in the country. The objective of this research was to investigate the oral health needs of schoolchildren at key ages, to inform future action.

**Materials and methods:**

This first national oral health survey of schoolchildren at 6-, 12- and 15-years was conducted in urban and rural settings across all four regions using a multi-stage cluster sampling in line with the WHO guidelines, adapted according to contemporary survey methods to include ‘International Caries Detection and Assessment System (ICDAS)’. Whilst parents were invited to complete a questionnaire for 6-year-old children, 12- and 15-year-olds self-completed a questionnaire. Data were weighted according to age and regional population and analysed using STATA v.15 and SPSS v.22.

**Results:**

A total of 1174 children participated across 22 schools from all four regions. Dental caries was prevalent (over 80% of all age-groups having *clinical decay*; ICDAS score ≥ 2) and largely untreated. No children had fillings and only 4% had missing teeth. Amongst 6, 12 and 15-year-olds, average decay levels at ICDAS > 3 threshold was 3.47 (primary teeth), 2.94 and 4.30 respectively. Almost, 10% (*n* = 119) of all children reported experiencing pain in their teeth with 7% (*n* = 86) children having PUFA lesions present. At least one in five children required one or more dental extractions. ‘Age’ was a significant predictor of dental caries experience and the odds of having dental caries experience was higher in rural areas at D_3–6_MFT (*p* < 0.05).

**Conclusion:**

The findings demonstrate a vast unmet oral health need in the children of SL. Using ICDAS as an epidemiological tool in a low-income country provides valuable insight to the pattern of oral disease to inform health service planning. Urgent action is required to address this silent epidemic.

## Introduction

Sierra Leone (SL) in West Africa has a population of over 7.5 million people, 42% of whom are aged below 15 years [1]. English is the official language; however, there are 15 local languages, amongst which *‘Mende, Temne and Krio’* are widely understood [1]. Only forty-four percent of the population are literate, and the average life expectancy is 57 years [1, 2]. The country ranks very low on the ‘human development index’ as 76% of the population live on less than $2 per day [2]. SL has not only suffered in the recent Covid-19 pandemic and Ebola epidemic in 2013–16, but emerged from a long civil war, 1991–2002 [3–7].

SL faces a critical shortage of oral care service delivery as there is scarcity of resources at all levels. It has only one hospital with a fully staffed dental clinic and approximately three whole-time equivalent public sector dentists for the whole country, with an additional 4–7 dentists working in private sector [8]. SL has a few dental auxiliaries (dental therapists/assistants/nurses) based in the capital Freetown [9]. SL also receives occasional visits from international dentists on short-term projects and organisations such as ‘Teethsavers International’, which conduct oral health promotion activities including basic preventive treatment [10]. SL does not have an approved national oral health strategy and having no dental training centre in the country, has historically relied on dentists and other dental personnel trained abroad [9].

There is a dearth of data on the oral health needs of the population with very few published, or unpublished, studies [11–14]. The first published study by Abdullah in the mid-1970’s reported major issues with respect to the organisation and delivery of dental services [15]. Since then, there have been several local studies in the districts of Freetown, Bo and Kenema [11, 12, 14]. Sten Normark, in the 1990s, prior to the civil war reported the prevalence of dental caries at 65% amongst 15-year-olds, and over 80% in adults aged 35–44 years, in both urban and rural areas of Bo and Kenema collectively [11, 12]. Similarly, a survey based on Freetown suggested a very high caries prevalence in 6-year-old school children (almost 80%) with mean DMFT (Decayed, Missing and Filled Teeth) scores of 7.2 for rural and 4.8 for urban localities [14]. Furthermore, epidemiological data on adults suggested the presence of considerable periodontal problems [11], compared with other African countries [16].

Thus, collectively, the findings of these published studies suggested considerable levels of dental caries in children (60–80%) and adults (around 80%) with some evidence of severe periodontal problems in adults. However, all these studies are cross sectional, almost three decades old, and mainly conducted prior to the civil war. Oral health can have a considerable impact on growth, nutrition, and the overall development of children; therefore, there is urgent need to explore the current prevalence of oral diseases, including dental caries and periodontal problems, in adults and especially children [17–20].

The aim of this research was to explore normative and perceived oral health needs and oral health behaviours across key age-groups of children, by type of area and region in SL.

## Materials and methods

This oral health survey, involving the three key age-groups of 6-, 12- and 15-year-olds, was carried out in line with agreed epidemiology methodologies globally [21], and in the United Kingdom (UK) [22, 23]. Ethical approval from King’s College London Research Ethics Committee (HR-15/16-1951; RESCMR-16/17-1951); and, thereafter, ‘Sierra Leone Ethics and Scientific Review Committee’ were obtained prior to conduct of the survey in 2017.

Considering the low levels of English literacy in SL [1], the research team worked with an artist, with the support of the research ethics committee, to develop a very simple information sheet and consent form utilising ‘pictorials’ to help explain the aim of this survey and the methods involved. The recruitment documents were developed following extensive discussions with local Sierra Leonean dental team members and colleagues with experience of similar research. The pictorial information sheet integrated the consent form in a simplified way to confirm participation in the study. Local support for this study was sought from the former Chief Dental Officer of SL and members of the ‘Teethsavers International’ group, who had experience of conducting dental camps at various sites across SL and were familiar with the schools and local area.

### Sampling for the survey

A multi-stage stratified cluster sample was used in line with the WHO guidelines for pathfinder surveys, this approach being particularly useful in countries such as SL which have limited data on oral health needs and where conduct of surveys is constrained by a variety of local factors [21]. SL consists of four regions comprising a total of 14 districts. Stage one of the sampling method involved selecting a capital district from each of the four regions (northern, eastern, southern and western area). Then within each of the capital cities, one primary and one secondary school were randomly selected with the aim being to survey at least 50 children in each age-group per district.

In the next stage, two large towns were randomly selected from the western (Freetown) and southern regions (Bo), considering practical feasibility in data collection and comparison with previous studies. In line with WHO methods [21], two sites were selected from each of the two large towns with one primary and one secondary school to be surveyed at each site. Subsequently, for rural populations, one village was randomly selected from each of the four regions, with one primary and one secondary school from each site. The WHO recommends sampling at least 25–50 subjects per age-group at each site, depending on the prevalence of disease (from low to high respectively) [21]. The validity of this sampling approach was tested and confirmed through experts in epidemiology and statistics before the survey [21].

### Consent and school recruitment process

The Ministry of Education, and the Ministry of Health and Sanitation in SL were contacted regarding the study, and they provided a list of schools (primary and secondary) for sampling purposes. For each site, a school was randomly selected through a computer-generated number. Following selection of a school, a courtesy call was used to verify the contact details of the school and establish an initial contact. If there was no contact established due to incorrect contact details from the list provided by the Ministry, a replacement school was randomly selected, again through a computer-generated number.

At the initial contact, by phone, the school head teachers were provided information regarding the study and an estimated time of visit. All the schools contacted by the research team accepted the invitation and agreed to take part in the survey on the scheduled date, except for one which wanted to schedule the survey on a different date.

The ‘information-consent form’ was sent at least one day in advance to the schools that agreed to participate. This was delivered by an advance party of the survey team. The head teachers at each school assisted in distribution of these information-consent forms to parents/guardians of children across all three age-groups.

For the 6-year-olds, written consent was sought from their parents prior to conduct of survey along with verbal consent of children on the day of the survey. For 12- and 15-year-olds, their parents were informed about the study and had the opportunity to withdraw their children (negative consent). On the day of the survey verbal and written consent were sought from the 12- and 15-year-old children personally. This approach of obtaining student consent was drawn from the protocol of the 2013 Children’s Dental Health Survey (CDHS) in the UK [22].

### Epidemiological tools

The diagnostic criteria for this survey were drawn from the UK national surveys (CDHS) [22, 23] for children examination using the ‘International Caries Detection and Assessment System (ICDAS)’ [24]. ICDAS is “a standardised system to inform decisions about appropriate diagnosis, prognosis, and clinical management of dental caries at both the individual and public health levels”, which takes account of the severity of dental caries [24]. Coding for dental caries was as follows: (D_2_) represents a *distinct visual change in enamel*; (D_3_) was *localised enamel breakdown*; (D_4_) represents *dentinal shadowing*; (D_5_) was *distinct cavity with visible dentine*; and (D_6_) *extensive cavity within visible dentine* [24]. Along with using ICDAS, the survey also explored the prevalence of untreated caries, trauma, and periodontal health.

### Training and calibration

The training and calibration exercises for the single examiner (SG), a doctoral student, were carried out in the UK in two stages. First, the student completed an online training exercise in dental caries and subsequently, attended a calibration session in one London school with a sample of 15 children. Second, following further training, an additional calibration session was conducted by an ICDAS gold standard examiner, using 191 printed clinical images, to assess inter-and intra-examiner reliability. The kappa scores for inter- and intra-examiner reliability were above 0.61.

### Questionnaire survey

To explore perceived oral health among children, a self-completion questionnaire survey was used. The instrument was drawn from national surveys within the UK and WHO methodology [21, 22, 25], and adapted for use in the local setting of SL. Having checked face validity with the dental team in-country, the questionnaire was initially piloted on a sample of children (*n* = 10) in the capital city of Freetown where King’s Health Partners (KHP) has a local team. The survey took no more than 15 min and was presented prior to the dental examination to maximise response [22]. The instrument covered the following topics:

- Self-rated dental and general health, including dental problems
- Satisfaction with appearance of teeth
- Impact of dental health on oral functioning
- Tooth brushing behaviour
- Visits to the dentist
- Barriers to attending for dental examinations
- Daily frequency of consumption of some food and drink categories
- Smoking and drinking behaviour.

### Survey process

On the day of the survey, the research team met with the school leaders and outlined the survey. Following this an oral health survey camp was set-up in the school environment. All children in the selected age-groups from each school were invited to participate. The school authorities assisted with running the survey and ensuring children presented in their relevant age-group and had provided consent. Children were briefed about the survey by the SL team in their local language (mainly *Mende, Temne and Krio*) and provided with an opportunity to clarify any issues or ask questions. The 12- and 15-year-old children, self-completed a questionnaire before undergoing the oral examination to minimise bias. For 6-year-old children, parents completed the questionnaire on behalf of their children when they visited the school site.

### Oral examination

All oral examinations were carried out in line with the survey protocol using the same portable chair under a standardised light source (under natural light). The survey was conducted over a six-week period in May–June 2017. The instruments used for the oral examination were single-use disposable plain mouth mirrors, whilst cotton wool rolls were used to remove debris and moisture, where necessary. All the clinical examinations were carried out by the same examiner (SG), supported by a local SL team member who recorded the findings on paper.

### Infection control

The examiner conducted oral examinations wearing Personal Protection Equipment (PPE) consisting of full body gown, gloves, face mask and a face visor in line with infection control practice at the main dental clinic at Connaught hospital in Freetown. This was in line with WHO [26], and the Centres for disease control and prevention (CDC) guidelines [27], which were in place during the Ebola virus disease outbreak between 2013 and 2016. Although during the conduct of the survey in 2017, SL was declared Ebola free [28], the research team continued to follow the guidelines for the safety of the researcher and the participants.

### Treatment referral

All the participating children who required basic dental treatment were assisted by the ‘Teethsavers International’ team. Routine extractions, where required, were undertaken by the dental team under the supervision of the lead dentist of Connaught hospital (former Chief Dental Officer of SL) who was part of this survey team. All children requiring moderate to advanced interventions received information on options of care available at Connaught hospital (Freetown) and other healthcare services in SL.

### Data entry and analysis

Data entry onto computer was carried out for both clinical and questionnaire data using EXCEL spreadsheets [29]. The data were cleaned, and a 20% sample was randomly selected to check for any inputting errors.

### Weighting the data

The data were weighted with respect to ‘age’ and ‘regional population’, according to the 2015 census data for SL [1]. The weighting was applied to the dataset used is both STATA and SPSS before conducting the analyses.

### Descriptive analyses

Following data cleaning, data were imported into STATA v.15 [30] and SPSS v.22 [31]. Descriptive analysis was carried out to report the characteristics of the sample such as age, gender, and the regional distribution of children in this study. To classify children according to urban and rural areas, the data from the SL census 2015 were used to inform the classification as advised by the Statistics SL team [1].

Proportions were also calculated for prevalence of dental caries at both tooth and surface level in both primary and permanent dentition, plaque, calculus, PUFA index, pain in mouth, trauma to teeth, and fluorosis along with items from the questionnaire survey. In addition, the mean number of teeth with decay (at different level of dental caries as per ICDAS coding), and missing teeth were also calculated.

### Univariate and bivariate analyses

To summarise and identify disease patterns, different levels of decay were examined by age, gender and region, a combination of univariate and bivariate analyses was used. A dmft/DMFT score was calculated for each child according to ICDAS decay codes from D_2_ to D_6_.

Thereafter, dmft/DMFT score was tested using chi-square test with independent variables such as age, gender, and region. These tests were carried out at each threshold level (from D_2_ to D_6_) and combining them for different ICDAS caries thresholds (D_2_ and above; D_3_ and above, etc.) to enable dental caries patterns to be examined in detail. All the p-values reported are not adjusted for multiple testing; and, hence, this should be considered when interpreting the results.

### Multivariate analyses

For multivariate analyses, items which were found to be statistically significant in univariate and bivariate analyses were incorporated into a logistic regression model. In this model dental caries experience (DMFT) was the dependent variable with independent variables such as age, sex, region, area, behavioural and quality-of-life-related items from the questionnaire. Variables which were not significant at the 10% level in bivariate analysis, were excluded. The two logistic models involved using the ‘type of area’ and ‘region’ variables separately. Model 1 included all significant variables with ‘Type of area’ and Model 2 included all significant variables with ‘region’.

## Results

### Survey participants: schools and schoolchildren

Out of the 22 participating schools, 13 were government, or government-aided; six were run by religious missionary groups; two were community supported schools; and one was privately owned. No head teacher declined to participate.

Out of the 1650 children approached across all four regions of SL, 71% (*n* = 1174) consented to be examined. As shown in Table 1, the majority were female (52%), and from an urban area (59%). The school environment appeared similar in nature across the four regions and by type of school (public, private, mission, community).

**Table 1:** Sierra Leone National Child Oral Health Survey participants, 2017 (*n* = 1,174).

Factors	Categories	6-year-olds % (*n*)	12-year-olds % (*n*)	15-year-olds % (*n*)	Total
**All Children**	**Total**	**37% (431)**	**22% (253)**	**42% (490)**	**100% (1,174)**
Sex	Male	51% (220)	45% (114)	46% (224)	48% (558)
	Female	49% (211)	55% (139)	54% (266)	52% (616)
Type of Area	Rural	41% (176)	46% (115)	38% (187)	41% (478)
	Urban	59% (255)	55% (138)	62% (303)	59% (696)
Region	Western	31% (133)	40% (101)	33% (161)	34% (395)
	Southern	29% (123)	32% (82)	29% (143)	30% (348)
	Eastern	20% (85)	14% (36)	23% (112)	20% (233)
	Northern	21% (90)	13% (34)	15% (74)	17% (198)

Schoolchildren from 22 schools in SL.

Bold values emphasize the total numbers.

### Dental caries experience in school children

Table 2 presents dental caries experience (dmft /DMFT) by age, sex, area and region for both deciduous and permanent dentitions. The most dramatic finding was the absence of any filled teeth amongst the children examined. Most dental caries was untreated; a small minority (2–6%) had missing teeth.

**Table 2:** Dental caries experience (dmft/DMFT) in children by age, gender, type of area and region in SL (*n* = 1174).

Characteristics		Age (yrs)	Cases (*n*)	D_2-6_MFT		D_3-6_MFT		D_4-6_MFT		D_5-6_MFT		D_6_MFT		Missing teeth (MT)		Filled teeth (FT)	
				%	Mean	%	Mean	%	Mean	%	Mean	%	Mean	%	Mean	%	Mean
All*		6^d^	431	84	4.65	72	3.47	51	1.71	28	0.76	16	0.38	6	0.14	0	0
		6	406	56	1.32	41	0.85	11	0.17	2	0.02	1	0.01	0	0	0	0
		12	253	83	4.34	65	2.94	40	1.04	21	0.4	15	0.24	2	0.06	0	0
		15	490	90	5.79	79	4.3	53	1.58	27	0.53	19	0.32	4	0.04	0	0
Sex	Female	6^d^	211	81	4.27	68	3.12	48	1.60	27	0.69	18	0.39	6	0.14	0	0
		6	198	50	1.14	35	0.69	6	0.11	1	0.01	0	0	0	0	0	0
		12	139	87	4.76	69	3.29	46	1.12	22	0.39	14	0.19	2	0.02	0	0
		15	266	91	5.98	79	4.32	54	1.65	28	0.54	19	0.33	3	0.04	0	0
	Male	6^d^	220	87	5	76	3.80	54	1.82	30	0.82	15	0.36	6	0.14	0	0
		6	208	61	1.49	47	1	15	0.22	4	0.04	2	0.02	0	0	0	0
		12	114	77	3.82	61	2.53	32	0.95	20	0.41	16	0.29	3	0.12	0	0
		15	224	89	5.56	80	4.28	52	1.49	26	0.53	18	0.30	4	0.05	0	0
Type of area	Urban	6^d^	255	83	4.50	73	3.36	49	1.60	28	0.76	17	0.43	5	0.14	0	0
		6	241	54	1.14	37	0.68	10	0.15	2	0.02	1	0.01	0	0	0	0
		12	138	78	4.01	63	2.78	42	1.04	25	0.41	17	0.30	4	0.12	0	0
		15	303	90	5.59	76	3.99	53	1.46	25	0.46	18	0.28	4	0.05	0	0
	Rural	6^d^	176	86	4.86	72	3.63	53	1.87	29	0.76	16	0.30	6	0.13	0	0
		6	165	58	1.57	48	1.10	12	0.19	3	0.03	1	0.01	0	0	0	0
		12	115	88	4.72	68	3.14	37	1.05	17	0.32	12	0.17	1	0.01	0	0
		15	187	90	6.12	84	4.81	52	1.76	30	0.66	20	0.38	3	0.04	0	0
Region*	East	6^d^	85	99	6.54	98	6.16	75	2.75	35	0.96	12	0.24	2	0.02	0	0
		6	75	85	1.95	84	1.83	19	0.35	1	0.01	0	0	0	0	0	0
		12	36	100	7.72	100	7.05	72	2.36	28	0.50	8	0.11	0	0	0	0
		15	112	100	7.94	99	7.13	79	2.88	31	0.62	11	0.18	2	0.02	0	0
	North	6^d^	90	94	5.18	87	4.01	69	2.37	37	1	18	0.42	2	0.02	0	0
		6	89	66	1.63	47	1.02	20	0.28	4	0.04	2	0.02	0	0	0	0
		12	34	100	5.08	76	3.26	38	1.41	18	0.35	9	0.12	0	0	0	0
		15	74	100	6.34	95	4.88	58	2.07	36	0.88	26	0.47	5	0.05	0	0
	South	6^d^	123	93	5.77	81	3.63	41	1.18	24	0.64	16	0.37	6	0.18	0	0
		6	115	70	1.83	50	0.97	7	0.11	3	0.03	1	0.02	0	0	0	0
		12	82	95	5.09	78	3.34	37	0.76	15	0.23	11	0.16	2	0.02	0	0
		15	143	98	6.64	87	4.88	41	1.02	16	0.29	13	0.17	1	0.01	0	0
	West	6^d^	133	59	2.03	38	1.23	32	1.10	22	0.57	19	0.44	10	0.26	0	0
		6	127	17	0.25	4	0.05	2	0.02	1	0.01	1	0.01	0	0	0	0
		12	101	60	2.27	39	1.05	32	0.68	26	0.44	22	0.39	4	0.15	0	0
		15	161	72	3.29	51	1.55	43	0.94	30	0.54	26	0.47	7	0.09	0	0

d = deciduous dentition.

*p < 0.05.

Dental caries was common in the primary dentition of 6-year-olds; 84% of children presenting with *clinical decay* (D_2_ and above). Children in the eastern region had a significantly higher dental caries experience score (*p* = 0.01) across all ICDAS caries thresholds, except at d_6_mft.

There was a clear trend by age in dental caries experience in the permanent dentitions of schoolchildren at all decay thresholds of ICDAS and was found to be highly significant (*p* = 0.01) at all four decay thresholds as shown (Table 2).

At regional level, the mean number of teeth with dental caries experience (dmft/DMFT) in all three age-groups was highest in the eastern region, followed by north, south and west respectively, at all decay thresholds of ICDAS (*p* = 0.01). The proportion of children with missing teeth was higher in the western region (includes the capital Freetown), but not significantly so.

### Other dental conditions

Table 3 presents the findings in relation to pain and PUFA lesions (Pulp, Ulceration, Fistula, Abscess) amongst children in the three key age-groups. One in eight 6-year-olds reported dental pain and approximately 1 in 10 children overall.

**Table 3:** Prevalence of pain in teeth and PUFA lesions seen in school children in SL, 2017 (*n* = 1174).

Age (yrs.)	Cases (*n*)	Pain reported		PUFA (Pulp, Ulceration, Fistula, Abscess)			
		No	Yes	Prevalence	No lesions	Single lesion	2 or more lesions
6	431	88.4% (*n* = 381)	11.6% (*n* = 50)	7% (*n* = 30)	93% (*n* = 401)	4.4% (*n* = 18)	2.5% (*n* = 12)
12	253	92.5% (*n* = 234)	7.5% (*n* = 19)	7% (*n* = 18)	92.8% (*n* = 235)	6.3% (*n* = 16)	0.7% (*n* = 2)
15	490	90.8% (*n* = 445)	9.8% (*n* = 45)	8% (*n* = 38)	92.2% (*n* = 452)	5.1% (*n* = 25)	2.6% (*n* = 13)

All PUFA lesions in 6-year-olds were seen related to their primary teeth.

The proportion of children at all age-groups with one or more PUFA lesions was in the region of 7–8%. All the PUFA lesions seen in children were of pulpal origin except one child each in the 6-and 12-year-old groups who had an abscess present.

Gingival health appeared normal for over 90% of all children (*n* = 1174); however, over 80% of all children had visible plaque (*n* = 1174) and over 70% of 12- and 16-year-olds had calculus present.

Dental trauma affected 0.5–2% of children, and mottling/fluorosis was very low with approximately 1% of all children affected.

### Health and oral health related quality of life

Self-reported data from the pupil questionnaire in Table 4 suggest that while many report their general and oral health as being good, 22% of 12-year-olds and 18% of 15-year-olds reported their dental health as ‘poor or ‘very poor’. Whilst general health was rated worse than oral health in both age-groups, the difference was not statistically significant.

**Table 4:** Descriptive findings for health and oral health related quality of life from pupil questionnaire for 12- and 15-year-olds.

Questionnaire themes	Categories	Sub-categories	12-year-olds % (*n*)	15-year-olds % (*n*)
Health related quality of life (HrQOL)	Overall general health	Very good/good	59% (130)	62% (253)
		Fair	17% (37)	16% (102)
		Very poor/poor	24% (52)	22% (139)
Oral health related quality of life (OHrQOL)	Overall dental health	Very good/good	66% (145)	67% (275)
		Fair	12% (26)	15% (92)
		Very poor/poor	22% (49)	18% (113)
	Dental problems in last 3 months	Sensitivity	27% (67)	27% (122)
		Toothache	19% (47)	22% (97)
		Bleeding/Swollen gums	16% (40)	18% (81)
		Broken tooth	5% (12)	8% (37)
		Ulcers	9% (23)	6% (29)
		Bad breath	6% (15)	5% (21)
		None	17%(43)	14% (64)
	Teeth aesthetics/appearance	Very satisfied/satisfied	62% (154)	67% (304)
		Neutral	14% (35)	14% (64)
		Very dissatisfied/dissatisfied	9% (52)	17% (75)
	Difficulty in eating	Not at all	49% (120)	53% (241)
		A little	30% (73)	25% (112)
		A lot/a fair amount	21% (51)	21% (94)
	Difficulty in speaking	Not at all	67% (166)	60% (277)
		A little	18% (45)	20% (88)
		A lot/a fair amount	13% (31)	18% (81)
	Difficulty in cleaning teeth	Not at all	57% (141)	63% (262)
		A little	22% (54)	26% (117)
		A lot/a fair amount	19% (46)	16% (70)
	Difficulty in relaxing/sleeping	Not at all	69% (171)	62% (280)
		A little	13% (33)	19% (85)
		A lot/a fair amount	15% (36)	80 (18%)
Oral health related quality of life (OHrQOL) Contd.	Felt upset/Irritated	Not at all	57% (140)	278 (62%)
		A little	24% (59)	97 (22%)
		A lot/a fair amount	16% (39)	14% (63)
	Difficulty in smiling	Not at all	62% (152)	66% (298)
		A little	21% (52)	21% (93)
		A lot/a fair amount	15% (36)	12% (56)
	Difficulty in completing school work	Not at all	68% (168)	61% (287)
		A little	14% (34)	22% (99)
		A lot/a fair amount	15% (36)	14% (62)
	Difficulty enjoying being with people	Not at all	63% (155)	67% (302)
		A little	15% (38)	20% (88)
		A lot/a fair amount	17% (42)	13% (57)
	Condition of teeth affected everyday life	Not at all	56% (139)	63% (286)
		A little	30% (73)	26% (118)
		A lot/a fair amount	12% (29)	8% (43)

The most reported condition, by 27% of 12- (*n* = 67) and 15- (*n* = 122) year olds, was having a sensitive tooth, followed by toothache, and bleeding or swollen gums. Only a minority reported not having experienced any dental problems in the previous three months (17% 12-year-olds; 14% 15-year-olds).

### Oral health behaviours (12 and 15-year-olds)

Most 12- (66%) and 15-year-olds (73%) reported ‘never having been to a dentist’, with only 8% having attended a check-up in both age-groups (Table 5). Attendance amongst 6-year-olds was lower, with only 3% (*n* = 8) of parents reporting their child had ever visited a dentist, and then only when they had a problem.

**Table 5:** Self-reported oral health and oral health behaviours of school children in SL.

Questionnaire themes	Categories	Sub-categories	12-year-olds	15-year-olds
Access to care	Visit to a dentist	Never been to a dentist	66% (163)	73% (327)
		Go only when I have trouble with teeth	19% (46)	17% (78)
		Went for check up	8% (19)	8% (38)
Oral hygiene	Brushing frequency	=1 time a day	33% (82)	32% (145)
		=2 times a day	26% (64)	32% (142)
		=3 times a day	23% (56)	17% (77)
		>3 times a day	13% (33)	15% (67)
		<1 time a day OR Never	2% (7)	4% (16)
	Brushing tool	Toothbrush	82% (202)	90% (365)
		Toothpick	8% (19)	2% (11)
		Thread	2% (6)	2% (7)
		Charcoal	2% (5)	2% (9)
		Other	3% (8)	3% (14)
	Use of toothpaste	Yes	90% (222)	87% (353)
		No	8% (20)	11% (52)
Diet	Sweets (e.g. chocolates)	= 4 times or more a day	13% (32)	8% (36)
		≤3 to 2 times a day	23% (57)	24% (110)
		≤1time a day	30% (73)	37% (168)
	Cakes or biscuits	=4 times or more a day	15% (37)	11% (51)
		≤3 to 2 times a day	34% (83)	38% (169)
		≤1 time a day	26% (64)	28% (118)
	Fruits	=4 times or more a day	28% (69)	23% (105)
		≤3 to 2 times a day	46% (113)	39% (222)
		≤1 time a day	16% (39)	23% (102)
	Fizzy drinks	=4 times or more a day	22% (55)	22% (100)
		≤3 to 2 times a day	15% (36)	19% (84)
		≤1 time a day	42% (103)	45% (204)
	Fruit juice	=4 times or more a day	11% (26)	10% (43)
		≤3 to 2 times a day	27% (67)	25% (114)
		≤1 time a day	32% (79)	37% (167)
	Water	=4 times or more a day	67% (165)	65% (292)
		≤3 to 2 times a day	9% (22)	12% (55)
		≤1 time a day	8% (19)	5% (18)
Other risk behaviours	Smoking	Never	80% (197)	90% (403)
		Tried sometimes	14% (34)	7% (34)
		Occasionally or Regularly	2% (5)	2% (8)
	Alcohol	Never	77% (189)	85% (381)
		Tried sometimes	14% (34)	12% (54)
		Occasionally or Regularly	6% (16)	3% (12)

In relation to diet, 22% of both 12-year (*n* = 55) and 15-year-olds (*n* = 100) reported having fizzy drinks four times a day or more, with 42% and 45% of 12-and 15-year-olds respectively having a fizzy drink at least once a day. Similarly, approximately 13% and 8% of both 12-and 15-year-olds reported having sweets four times a day or more; and almost one third of 12-year-olds and 40% of 15-year-olds reporting having them at least once a day.

Most children at both 12- (95%) and 15-years (96%) reported brushing their teeth once a day, or more, with 26% and 32% respectively brushing twice a day. A majority of 12- (82%) and 15-year-olds (90%) reported using a toothbrush and toothpaste, with the remainder using other products such as toothpicks, thread and charcoal. Similarly, most parents of 6-year-olds also reported that their children used toothbrush (98%) and toothpaste (96%). There was some evidence of tobacco and alcohol use by older children.

### Predictors of dental caries experience in children

The logistic regression model included age, sex, type of area and region as predictor variables, whereas items from the questionnaire were excluded. As expected, the odds of dental caries experience significantly increased by age (*p* < 0.01) at all ICDAS thresholds; however, there was no significant difference by sex (Table 6). ‘Region’ (*p* < 0.01) was a significant predictor of dental caries experience across all thresholds, and ‘urban/rural’ area was only significant at D_3-6_MFT, with rural areas having higher caries experience.

**Table 6:** Logistic linear models using dental caries experience in permanent teeth at different caries threshold by type of area (Model 1) and region (Model 2).

Predictors (Reference Category)		D_2-6_MFT		D_3-6_MFT		D_4-6_MFT		D_5-6_MFT	
		MODEL 1 with ‘Type of Area’ OR (95% CI)	MODEL 2 with ‘Region’ OR (95% CI)	MODEL 1with ‘Type of Area’ OR (95% CI))	MODEL 2 with ‘Region’ OR (95% CI)	MODEL 1 with ‘Type of Area’ OR (95% CI)	MODEL 2 with ‘Region’ OR (95% CI)	MODEL 1with ‘Type of Area’ OR (95% CI)	MODEL 2 with ‘Region’ OR (95% CI)
Sex (Female)	Male	1.02 (0.76–1.38)	0.75 (0.53–1.07)	1.09 (0.84–1.42)	1.00 (0.73–1.36)	0.95 (0.73–1.24)	0.96 (0.73–1.27)	0.97 (0.70–1.35)	0.99 (0.71–1.38)
Age (6-year-olds)	12-year-olds 15-year-olds	3.76 (2.57–5.50) * 7.41 (5.18–10.60) *	10.06 (6.05–16.73) * 17.72 (10.94–28.69) *	2.67 (1.92–3.71) * 5.62 (4.17–7.57) *	5.83 (3.83–8.88) * 11.59 (7.81–17.21) *	5.60 (3.73–8.39) * 9.51 (6.62–13.67) *	7.32 (4.77–11.23) * 11.22 (7.65–16.45) *	11.90 (5.76–24.62) * 16.45 (8.25–32.82) *	12.76 (6.14–26.51) * 17.10 (8.55–34.21) *
Type of area (Urban)	Rural	1.24 (0.92–1.68)	n/a	1.52 (1.17–1.98) *	n/a	0.97 (0.74–1.27)	n/a	1.08 (0.78–1.51)	n/a
Region (East)	North South West	n/a n/a n/a	0.36 (0.17–0.78) 0.34 (0.16–0.71) * 0.01 (0.01–0.04) *	n/a n/a n/a	0.14 (0.07–0.28) 0.12 (0.06–0.23) * 0.01 (0.01–0.02) *	n/a n/a n/a	0.48 (0.30–0.75) 0.23 (0.15–0.34) * 0.20 (0.14–0.31) *	n/a n/a n/a	1.12 (0.67–1.89) 0.47 (0.28–0.77) * 0.92 (0.60–1.43)

*n/a* not applicable, *OR* odds ratio, *CI* confidence interval, Permanent dentition.

*p < 0.05.

## Discussion

This study was the first to explore oral health at a national level in SL and helped to provide baseline data on dental caries experience in school children along with their periodontal health, self-reported oral health problems and oral hygiene behaviours. Most schoolchildren examined had *clinical* decay, with almost a quarter of children having *extensive* dental decay in tooth which eventually could result in tooth loss. Almost one out of 10 of all children reported pain and surprisingly no teeth with any sort of dental restorations were seen in this survey.

### Dental caries experience

A stark finding from this study was that most children had untreated dental caries. Only a minority (between 10% and 17%) were found to have no dental caries across the three age-groups examined. This is in contrast with epidemiological evidence from the African region where levels of dental caries were traditionally reported as low compared with the global average [32–34].

The findings may be compared with the 2013 UK CDHS which used a comparable approach [23, 35, 36]. The 2013 CDHS reported that 34% of 12-year-olds and 46% of 15-year-olds had *obvious* decay experience (D_4-6_MFT) [23, 35, 36], whereas in SL it was slightly higher at 40% and 53% respectively. Younger children were not directly comparable by age with 31% of 5-year-olds in the UK [23, 35, 36] and 51% 6-year-olds in SL having *obvious* decay experience in their primary dentition, nonetheless, suggesting caries levels were higher in SL.

A very notable finding from the survey was the absence of any filled teeth amongst the children examined. Only a minority of children across all three age-groups had missing teeth which reflects that most dental caries was untreated. This fact is substantiated by the findings from the qualitative study in which SL dental professionals expressed their concern regarding people’s choice in which they rarely prefer restorative treatment and but favour urgent treatment care such as extractions which are cheaper than restorations [37]. However, the preference for extraction of teeth could also be related to the fact that people attend dental services mainly for urgent care, in desperation because other approaches have failed, and their condition may be life-threatening. There is similar evidence from sub-Saharan Africa (Tanzania) that uptake of restorative treatment (mainly fillings) is very low, and patients’ preference is for tooth extraction [38, 39]. Absence of restorations may also be attributed to the fact that most dental professionals are based in the capital city of Freetown and therefore, access to restorative treatment is not feasible. Additionally, as highlighted in our earlier research [37], people’s previous traumatic dental experiences may inhibit them accessing dental care.

### Regional difference in caries experience

The findings from the study also suggest that the prevalence of dental caries experience was higher in the eastern region of SL. It is difficult to formulate a hypothesis as no significant difference was reported with respect to diet or oral hygiene behaviours by region. The role of dietary sugar is well documented in literature [40–43]; however, field notes suggest that the availability of free sugars in the school environment was similar where the school lunch table looked more like a cariogenic a ‘tuck shop’. The protective effect of fluoride in caries prevention is known and whilst data from an old SL study [12], highlight low levels of fluoride in the eastern region; however, it wasn’t exclusive just for that region. Most of the population in SL is economically poor and socio-economic indicators across all four regions are fairly similar [1]. However, the Eastern region is remote, mostly hilly, and isolated compared to other three regions of SL. Also, there is possibility that there could be some other region-specific characteristics which were not spotted during the fieldwork. For instance, an ad-hoc study in 1995 conducted in a remote village in the Eastern region suggested that consumption of bush fruit which was high in fructose content could be a potential factor for dental caries [44]. However, all these findings from ad-hoc studies in SL along with the anecdotal evidence should be interpreted with caution as they may not necessarily be representative of that region.

### Oral health behaviour

The questionnaire findings suggested that most children report using a toothpaste and toothbrush at least once a day. The clinical examination, however, revealed a high prevalence of visible plaque and calculus in SL school children. These findings are strikingly similar to the study conducted by Normark in SL in 1991 [11]. Normark reported that 95% (*n* = 128) of 15-year-olds reported brushing teeth using a toothbrush and toothpaste at least once a day or more, which compares with 96% (*n* = 390) of 15-year-olds currently. Similarly, the presence of visible plaque was reported in 92% of 15-year-olds in 1991 compared with 95% currently. Whereas almost 90% of 15-year-olds in 1991 were reported to have supragingival (visible) calculus and it was slighlty lower at 85% in the current study. Similar results have been reported from studies in LICs such as Tanzania and Ghana wherein children who brushed daily still had a considerable amount of plaque [45, 46]. Observationally, the availability of toothbrushes and toothpaste within SL appears to vary and they are less apparent outside the capital districts of each region.

In relation to diet, almost 4 out of 10 children reported consuming sweets and fizzy drinks at least once a day. Similar findings were reported by Normark with almost 37% of 15-year-olds reporting consuming sweets at least once a day [11]. Furthermore, in both studies there was no significant difference between urban and rural areas. Interestingly SL children reported consuming slightly higher levels of sugary drinks (22% for both) compared with their counterparts in the UK (16%; 14%) at 12- and 15-years of age respectively.

There are concerns amongst health professionals that post-civil war availability of cheap sugar is evident [37]. This is in line with the view that the shift in prevalence of dental caries could be associated with the geo-political race globally wherein high-income countries are seen actively involved with LICs to have economic control, which in turn opens up markets for leading companies offering tobacco, alcohol, sugary and high fat-saturated products at very cheap prices [47].

Furthermore, a range of potential factors could have influenced the self-reported behaviours which is difficult to establish. Consideration should be given to the fact that these children responded to the questionnaire in a school setting and therefore, the influence of peers or teachers could result in a potential bias. These children are not used to completing questionnaires or answering dental questions, thus, although studying in English, language could have been a potential issue which affected participating children.

### Dental caries thresholds

The advantage of the ICDAS epidemiological tool, now termed as “International Caries Classification and Management System (ICCMS)”, is that it facilitates comparison across past surveys which have used WHO methodology [48–50], and allows dental caries patterns to be profiled. However, this highlights the importance of determining a caries threshold for comparison of data.

**Fig. 1:**
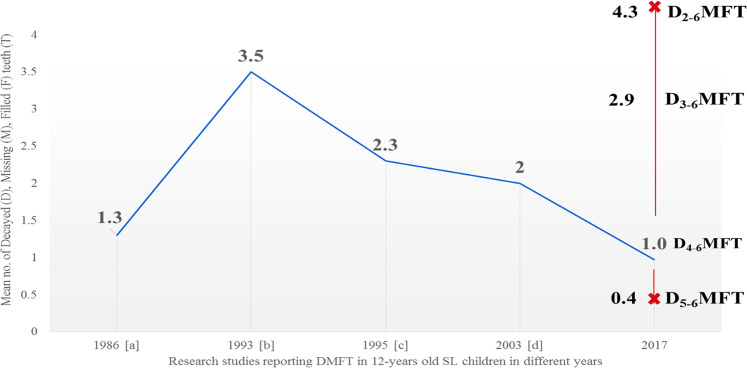
Trend of dental caries experience (DMFT) in 12-year-olds in SL from 1986 to 2017. **a** Woodward and Walker [41] (1994). **b** Nörmark [12] (1993). **c** Bly and Bradnock [44] (1995). **d** Don-Davis [14] (2003). 2017 = findings from the current study.

Figure 1 demonstrates this challenge when we compare findings from our study with past surveys which have been conducted in SL. It presents mean DMFT scores for 12-year-olds from cross sectional local studies since 1986 and compares them with our latest study. There is, however, insufficient data from past research to be clear about the caries threshold utilised. Thus, depending on the threshold selected, the mean caries experience for 12-year-olds from this study either increases or decreases when compared to past surveys. For example, if we consider D_**3**_ threshold, the mean caries experience of the current cohort of 12-year-olds in SL is higher than that reported in 2003.

Several studies have addressed this issue of determining the caries threshold for comparison [51–53]. Whilst some may argue that the D_3_ threshold has been deemed most suitable; however, others take contrasting views [54]; hence, the desire of the authors to present the full caries profile for each age-group. Furthermore, caries patterns have changed over the last five to six decades [55–57], and we suggest that developments in epidemiological tools are needed to monitor the caries process more accurately and include early caries, whilst distinguishing it from *obvious* and *extensive* decay. This study, therefore, highlights this issue and the importance of identifying the caries threshold for comparing data universally.

### Strengths and limitations

The fieldwork for the oral health survey was a challenge considering the logistic issues in SL. However, advanced careful planning and partnership enabled the fieldwork to be carried out as intended. As participation in the survey involved children and parents, gaining appropriate informed consent was crucial. This was achieved by using a basic information sheet with pictures to explain about the survey, given that many will never have been examined by a dentist. In addition, the local SL team was fluent in all local SL languages and assisted when informing participants about the study.

Although the standard of English across all schools in SL was not uniform, it was sufficient as it is the medium of delivering teaching; hence, the decision to conduct the questionnaire survey in English. Children, parents, and teachers were provided with a supplemental verbal translation by the local SL team. As there were limited current data on schoolchildren, using the WHO basic pathfinder sampling method [21], was deemed appropriate for determining baseline oral needs. In addition, the study was unique in demonstrating the potential to use the ICCMS epidemiological tool in a low-resource setting which has enabled oral health workforce requirements to be estimated and published [58]. This is particularly important as urgent action is needed to develop dental workforce and strengthen the fragile dental system in line with WHO guidance on Oral health, Health workforce development and Universal Health Coverage (UHC) working in partnership with other agencies [59–62], and delivering ‘*Optimal Oral Health for All’* [63].

## Conclusion

In summary, our findings demonstrate that using ICCMS (formerly ICDAS) as an epidemiological tool in a low-income country provides valuable insight to the pattern of oral disease to inform health service planning and reveals stark oral and dental needs in SL. Given the granularity of this epidemiological data, they have provided a sound foundation to model future workforce options which are currently under consideration in SL. Urgent action is required to address this silent epidemic.

## References

[1] Statistics Sierra Leone. 2015 Population and Housing Census. 2016. https://www.statistics.sl/wp-content/uploads/2017/01/final-results_-2015_population_and_housing_census.pdf. Accessed 15 Feb 2017.

[2] The World Bank. Sierra Leone-World Development Indicators. 2013 http://data.worldbank.org/country/sierra-leone#cp_wdi. Accessed 2 Aug 2013.

[3] Médecins Sans Frontières. Access to healthcare in post-war Sierra Leone. Summary of a 2005 survey in four districts: Kambia, Tonkolili, Bombali, Bo. Médecins Sans Frontières; 2006.

[4] Briand S, Bertherat E, Cox P, Formenty P, Kieny M-P, Myhre JK (2014). The International Ebola Emergency. N Engl J Med.

[5] The Lancet. Ebola in west Africa: getting to zero. Lancet. 2015;385:578. 10.1016/S0140-6736(14)62478-8 25682525

[6] Dallatomasina S, Crestani R, Sylvester Squire J, Declerk H, Caleo GM, Wolz A (2015). Ebola outbreak in rural West Africa: epidemiology, clinical features and outcomes. Tropical Med Int Health.

[7] Buonsenso D, Cinicola B, Raffaelli F, Sollena P, Iodice F (2020). Social consequences of COVID-19 in a low resource setting in Sierra Leone, West Africa. Int J Infect Dis.

[8] World health Organisation [WHO]. Towards health systems resilience: Strategic investments for a needs-based health workforce in Sierra Leone. 2015. Contract No.: 28 August.

[9] Gallagher JE, Challacombe SC. Oral Health – a Silent Emergency. Initial Report from Sierra Leone. London: King’s College London; 2013.

[10] Teethsavers International. Home. 2015. http://www.teethsavers.org/index.php.

[11] Nørmark S (1991). Oral health among 15-and 35-44-year-olds in Sierra Leone. Tandlaegebladet.

[12] Nörmark S (1993). Social indicators of dental caries among Sierra Leonean schoolchildren. Eur J Oral Sci.

[13] Koroma TT. Impact of oral health on everyday living of people aged 65 years and above living in Waterloo, Sierra leone. London: King’s College London; 2011.

[14] Don-Davis P. Oral Health Status of 6 and 12 Year Old School Children in Freetown, Sierra Leone. South Africa: University of Stellenbosch; 2003.

[15] Abdullah AS (1975). The state of dental health and oral hygiene in Sierra Leone. J Am Soc Psychosom Dent Med.

[16] Baelum V, Scheutz F (2002). Periodontal diseases in Africa. Periodontology 2000.

[17] Sheiham A (2006). Dental caries affects body weight, growth and quality of life in pre-school children. Br Dent J.

[18] Do LG, Spencer A (2007). Oral health‐related quality of life of children by dental caries and fluorosis experience. J Public Health Dent.

[19] Gerritsen AE, Allen PF, Witter DJ, Bronkhorst EM, Creugers N (2010). Tooth loss and oral health-related quality of life: a systematic review and meta-analysis. Health Qual Life Outcomes.

[20] Low W, Tan S, Schwartz S (1998). The effect of severe caries on the quality of life in young children. Pediatr Dent.

[21] World Health Organisation (WHO). Oral Health Surveys. Basic methods. 5 ed. Geneva: WHO; 2013. http://apps.who.int/iris/bitstream/handle/10665/97035/9789241548649_eng.pdf;jsessionid=30B0B58FBFB3C634AC4D8CF0DB9C7D41?sequence=1. Accessed 18 May 2018.

[22] Office for National Statistics, Health and Social Care Information Centre, Research Consortium. UK Child Dental Health Survey 2013: protocol and tools. United Kingdom: Health and Social Care Information Centre; 2013.

[23] Pitts NB, Chadwick B, Anderson T Report 2: Dental Disease and Damage in Children England, Wales and Northern Ireland. London: Health and Social Care Information Centre; 2015.

[24] Pitts N, Ekstrand K, Foundation TI (2013). International Caries Detection and Assessment System (ICDAS) and its International Caries Classification and Management System (ICCMS) – methods for staging of the caries process and enabling dentists to manage caries. Community Dent Oral Epidemiol.

[25] Sullivan I, Lader D, Beavan-Seymour C, Chenery V, Fuller E, Sadler K. Foundation report: Adult Dental Health Survey 2009 (Technical information). United Kingdom: The Health and Social Care Information Centre; 2011.

[26] World Health Organisation (WHO). Clinical care for survivors of Ebola virus disease. 2016. http://www.who.int/csr/resources/publications/ebola/guidance-survivors/en/. Accessed 08 August 2018.

[27] Centres for Disease Control and Prevention (CDC). Infection Prevention and Control Recommendations for Hospitalized Patients Under Investigation (PUIs) for Ebola Virus Disease (EVD) in U.S. Hospitals. 2015. https://www.cdc.gov/vhf/ebola/clinicians/evd/infection-control.html. Accessed 08 August 2018.

[28] World Health Organisation (WHO). Statement on the end of the Ebola outbreak in Sierra Leone. 2015. http://www.afro.who.int/en/sierra-leone/press-materials/item/8140-statement-on-the-end-of-the-ebola-outbreak-in-sierra-leone.html.

[29] Microsoft. Excel 2016. USA: Microsoft; 2016.

[30] StataCorp. Stata Statistical Software: Release 15. 15 ed. College Station, Texas: StataCorp LLC; 2017.

[31] IBM Corp. IBM SPSS Statistics for Windows, Version 22.0. 22 ed. Armonk, NY: IBM Corp; 2013.

[32] African Health Observatory. WHO African region. 2015. http://www.aho.afro.who.int/profiles_information/images/6/6c/SL_Table_9.PNG.

[33] Petersen PE, Bourgeois D, Ogawa H, Estupinan-Day S, Ndiaye C (2005). The global burden of oral diseases and risks to oral health. Bull World Health Organ.

[34] World Health Organisation [WHO]. World Health Statistics 2015. 2015. http://www.who.int/gho/publications/world_health_statistics/2015/en/.

[35] Vernazza CR, Rolland SL, Chadwick B, Pitts N (2016). Caries experience, the caries burden and associated factors in children in England, Wales and Northern Ireland 2013. Bdj.

[36] Wang X, Bernabe E, Pitts N, Zheng S, Gallagher JE (2021). Dental caries thresholds among adolescents in England, Wales, and Northern Ireland, 2013 at 12, and 15 years: implications for epidemiology and clinical care. BMC Oral Health.

[37] Ghotane SG, Challacombe SJ, Gallagher JE. Fortitude and resilience in service of the population: a case study of dental professionals striving for health in Sierra Leone. BDJ open. 2019;5:7. 10.1038/s41405-019-0011-2 PMC6513870 31098298

[38] Nyamuryekung’e KK, Lahti SM, Tuominen RJ. Attitudes towards tooth fillings in Tanzanian adults and its association with previous filling experience. BMC Oral Health. 2018;18:12. 10.1186/s12903-018-0474-x PMC5774145 29347931

[39] Kikwilu E, Frencken J, Mulder J, Masalu J (2009). Barriers to restorative care as perceived by dental patients attending government hospitals in Tanzania. Community Dent Oral Epidemiol.

[40] Steyn N, Myburgh N, Nel J (2003). Evidence to support a food-based dietary guideline on sugar consumption in South Africa. Bull World Health Organ.

[41] Woodward M, Walker AR (1994). Sugar consumption and dental caries: evidence from 90 countries. Br Dent J.

[42] Sheiham A, James W (2015). Diet and dental caries: the pivotal role of free sugars reemphasized. J Dent Res.

[43] Burt BA, Pai S (2001). Sugar consumption and caries risk: a systematic review. J Dent Educ.

[44] Bly S, Bradnock G (1995). Dental problems of a rural community in Sierra Leone. Community Dent Health.

[45] Addo-Yobo C, Williams S, Curzon M (1991). Oral hygiene practices, oral cleanliness and periodontal treatment needs in 12-year old urban and rural school children in Ghana. Community Dent Health.

[46] Nörmark S, Mosha HJ (1989). Relationship between habits and dental health among rural Tanzanian children. Community Dent Oral Epidemiol.

[47] Smith R, Exeter C. Report of the non-communicable disease working group. United Kingdom: The Global Health summit; 2012.

[48] Altarakemah Y, Al-Sane M, Lim S, Kingman A, Ismail AI (2013). A new approach to reliability assessment of dental caries examinations. Community Dent Oral Epidemiol.

[49] Jablonski-Momeni A, Stachniss V, Ricketts DN, Heinzel-Gutenbrunner M, Pieper K (2008). Reproducibility and Accuracy of the ICDAS-II for Detection of Occlusal Caries in vitro. Caries Res.

[50] Richards D (2005). Outcomes, what outcomes?. Evid. Based Dent..

[51] Braga MM, Oliveira LB, Bonin GAVC, Bönecker M, Mendes FM (2009). Feasibility of the International Caries Detection and Assessment System (ICDAS-II) in Epidemiological Surveys and Comparability with Standard World Health Organization Criteria. Caries Res.

[52] Mendes FM, Braga MM, Oliveira LB, Antunes JL, Ardenghi TM, Bonecker M (2010). Discriminant validity of the International Caries Detection and Assessment System (ICDAS) and comparability with World Health Organization criteria in a cross-sectional study. Community Dent Oral Epidemiol.

[53] Clara J, Bourgeois D, Muller-Bolla M (2012). DMF from WHO basic methods to ICDAS II advanced methods: a systematic review of literature. Odonto-stomatologie tropicale Tropical Dent J.

[54] Raveen Haricharan Bhoopathi PUP, Vinayak Kamath B, Gopal Deepika, Kumar Sai, Kulkarni Ganesh (2017). Caries detection with ICDAS and the WHO criteria: a comparitive study. J Clin Diagnostic Res..

[55] White DA, Tsakos G, Pitts NB, Fuller E, Douglas GVA, Murray JJ (2012). Adult Dental Health Survey 2009: Common oral health conditions and their impact on the population. Br Dent J.

[56] Petersson GH, Bratthall D (1996). The caries decline: a review of reviews. Eur J Oral Sci.

[57] Pieper K, Schulte AG (2004). The decline in dental caries among 12-year-old children in Germany between 1994 and 2000. Community Dent Health.

[58] Ghotane SG, Don-Davis P, Kamara D, Harper PR, Challacombe SJ, Gallagher JE (2021). Needs-led human resource planning for Sierra Leone in support of oral health. Hum Resour Health.

[59] UN General Assembly. Universal health coverage: moving together to build healthier world. 2019. https://www.un.org/pga/73/wp-content/uploads/sites/53/2019/07/FINAL-draft-UHC-Political-Declaration.pdf. Accessed 18 May 2021.

[60] World Health Organization (WHO). Oral health. Executive Board Resolution EB148/R1 (21 January 2021). 2021. https://apps.who.int/gb/ebwha/pdf_files/EB148/B148_R1-en.pdf. Accessed 18 October 2021.

[61] World Health Organization (WHO). Oral Health. Achieving better oral health as part of the universal health coverage and noncommunicable disease agendas towards 2030. Report by the Director-General (EB148/8) 148th Session of the Executive Board, Provisional Agenda Item 6. 2020. https://apps.who.int/gb/ebwha/pdf_files/EB148/B148_8-en.pdf. Accessed 18 October 2021.

[62] Global Health Workforce Alliance (GHWA), World Health Organisation (WHO). Health Workforce 2030: a global strategy on human resource for health. Geneva: World Health organisation; 2014. http://www.who.int/workforcealliance/knowledge/resources/strategy_brochure9-20-14.pdf?ua=1.

[63] Glick M, Williams DM (2021). FDI vision 2030: delivering optimal oral health for all. Int Dent J.

